# Bioactivity of *Malva Sylvestris* L., a Medicinal Plant from Iran

**Published:** 2011

**Authors:** Seyed Mehdi Razavi, Gholamreza Zarrini, Ghader Molavi, Ghader Ghasemi

**Affiliations:** 1 *Department of Biology, Faculty of Sciences, University of Mohaghegh Ardabili, Ardabil, Iran*; 2 *Department of Animal Sciences, Faculty of Natural Sciences, University of Tabriz, Tabriz, Iran*; 3 *Department of Statistics, Faculty of Science, University of Mohaghegh Ardabili, Ardabil, Iran*

**Keywords:** Antibacterial, Antifungal, Cytotoxicity, Malva sylvestris

## Abstract

**Objective(s):**

*Malva sylvestris* L. (Malvaceae), an annual plant, has been already commonly used as a medicinal plant in Iran. In the present work, we evaluate some bioactivities of the plant extracts.

**Materials and Methods:**

The aired-dried plant flowers and leaves were extracted by soxhlet apparatus with n-hexane, dichloromethane and methanol. The antimicrobial, cytotoxic, and phytotoxic of the plant extracts were evaluated using disk diffusion method, MTT, and Lettuce assays, respectively.

**Results:**

Both flowers and leaves of *M. sylvestris *methanol extracts exhibited strong antibacterial effects against *Erwinia carotovora*, a plant pathogen, with MIC value of 128 and 256 µg/ml, respectively. The flowers extract also showed high antibacterial effects against some human pathogen bacteria strains such as *Staphylococcus aureus*, *Streptococcus agalactiae*, *Entrococcus faecalis*, with MIC value of 192, 200 and 256 µg/ml, respectively. The plant methanol extracts had relatively high cytotoxic activity against MacCoy cell line.

**Conclusion:**

We concluded that *Malva sylvestris* can be candidated as an antiseptic, a chemopreventive or a chemotherapeutic agent.

## Introduction

Genus *Malva* L. (Malvaceae) is represented by 40 taxa in all over of the world. *Malva sylvestris* L. is an annual plant with shallowly lobed leaves and purple flowers which bloom in late spring. This plant is native to Europe, North Africa and South-west Asia. The plant prefers damp areas, such as the ocean, salt marshes, meadows, sides of ditches and banks of tidal rivers (1). 

*M. sylvestris* is commonly used as vegetable and a medicinal plant in Iran where it is named as Panirak. The plant flowers are used as a remedy for cut wound, eczema, dermal infected wounds, bronchitis, digestive problems, and inflammations (2). Regarding the results of wang (2005), anthocyanins from *M. sylvestris* caused decreases in total cholesterol and triglycerides of plasma. It is also shown that the extracts of some *Malva* species protected rats from gastric lesions induced by ethanol. This antiulcerogenic activity may be associated with the high mucilage content from the plant species (3).

There are many reports on phytochemicals from *M. sylvestris*. Some reports revealed the prescence of malvone A, a naphthoquinone and different known monoterpenes, aromatic compounds, and a tetrahydroxylated acyclic diterpense (4,5).

In the present work, we evaluated cytotoxic, phytotoxic, and antimicrobial activities of different plant extracts.

## Materials and Methods

***Plant materials***

The flowers and leaves of *M. sylvestris* were collected from around Tabriz, Iran during June 2009. A voucher specimen was deposited at the Herbarium of faculty of Sciences, University of Mohaghegh Ardabili, Ardabil, Iran (No: 1389-2).

***Preparation of the extracts***

Air-dried plant flowers and leaves were extracted using a soxhlet type apparatus with n-hexane, dichloromethane, and methanol, respectively. The extracts were dried in vacuum.

***Antibacterial assay***

The antibacterial activities of the plant extracts were determined against *Escherichia coli* (PTCC 1047), (Persian Type Culture Collection) *Staphylococcus aureus* (PTCC 1112), *Entrococcus faecalis* (PTCC 1190), *Streptococcus agalactiae* (PTCC 1321), *Erwinia carotovora* (PTCC 1675), and *Staphylococcus aureus* (E_38_) by the disc diffusion method (Razavi *et al*, 2009 b). Muller- Hinton agar (MHA) (Oxoid) was used for preparation of the media of bacteria. The filter paper discs (6 mm in diameter) were individually impregnated with 15 μl of stock solution of the plant extracts (1.5 mg/ml) and then placed onto the agar plates which had previously been inoculated with the tested microorganisms. The plates were inoculated at 37 °C for 24 hr. The diameters of inhibition zones were measured in millimeters. All the tests were performed in duplicate. Gentamicin (10 μg) and erythromycin (15 μg) served as positive control. The MICs of the extracts against the test microorganisms were determined by the Agar dilution method (6).

***Antifungal assay***

The antifungal activities of the plant extracts were determined against *Candida kefyr *(ATCC 3896),* Candida albicans* (ATCC 14053),* Aspergillus niger* (PLM 1140), *Pinicillum SP,* and *Sclerotinia sclerotiorum *by the disc diffusion method (Lorain 1996). Sabouraued dextrose agar (SDA) (Oxoid) was used for preparation of the media of the fungal strains. The filter paper discs (6 mm in diameter) were individually impregnated with 15 μl of stock solution of the extracts (1.5 mg/ml) and then placed onto the agar plates which had previously been inoculated with the tested microorganisms. Amphotericin B (10 μg) disc was applied as positive control and the plates were inoculated with the fungi incubated at 30 °C for 48 hr. The diameters of inhibition zones were measured in millimeters. All the tests were performed in duplicate. The MICs of the extracts against the test microorganisms were determined by the agar dilution method (6).

***Cell culture conditions and cytotoxic assay***

MacCoy cell lines (Pasteur, C_123_) were grown in 

RPMI 1640 (Gibco, No 51800-019) medium. Each 500 ml of the medium consisted of 5.2 g RPMI powder, 1 g of sodium bicarbonate, 1% W/V of penicillin/streptomycin and supplemented with 10% heat- inactivated fetal calf serum (FCS) in demonized water (7). Completed medium was sterilized by filtering through 0.22 μm microbiological filters (Art no 11107-25). Cell line was maintained in a humidified atmosphere of 5% CO_2_ at 37 °C in an incubator. The stock solutions of methanol extracts of *M. sylvestris *flowers and leaves were prepared by dissolving the compound in 100 μl DMSO (1 mg/ml). The final concentration of the extract was 0.70, 0.50, 0.30, 0.3, 0.10, and 0.05 mg/ml. Cells were plated in the appropriate media on 24-well microplates in a 500 μl total volume at a density of 6×10^5 ^cell/ml. Three different tests were performed for each concentration to validate the results. The plates were incubated at 37 °C in 5% CO_2_ for a time course of 16 hr. For evaluating cell viability, the media were removed and 50 μl of a 5 mg/ml solution of MTT was added to each treated cell culture. The cells were incubated for 3 hr at 37 °C in 5% CO_2_ and the foramazone crystals were dissolved in 1 ml DMSO until the color reaction became uniform. The optical density was determined at 570 nm using a spectrophotometer. The amount of MTT converted to formazan is a sign of the number of viable cells. Media- only treated cells served as the indicator of 100% cell viability. The 50% inhibitory concentration (IC_50_) was defined as the concentration that reduced the absorbance of the untreated wells by 50% of the control in the MTT assay. Viability percentage was evaluated as OD _treatment_/OD _control_ (8).

***Phytotoxic assay***

Lettuce** (***Lactuca sativa* L. CV. Varamin**) **seeds were used to test germination response to different concentration of the plant root extracts. The stock solutions of the methanol extract were prepared by dissolving the extracts in the minimum volume of dimethylsulphoxide (DMSO). The stock of methanol extract of *M. sylvestris* leaves was obtained by dissolving in the sterile water. Different concentration of extracts (1, 0.1, 0.01, 0.001 mg/ml) were obtained by dilution with deionized water. Parallel controls were performed with the same volume of DMSO. All seeds were surface sterilized with sodium hypochloride (1%). Four replicates, each of 25 seed, were prepared for each treatment using sterile Petri dishes (90 mm) lined with one sterile filter paper (Whatman, number 2). Five ml of different concentration of the extracts was added to each Petri dish. Prepared plates were then placed in a germination cabinet at 25 °C in the dark. Germination was deemed to occur only after the radicle had protruded beyond the seed coat by at least 1 mm. After 1 week, in the each treatment, germination percentage was determined (9,10).

***Statistical analysis***

In all assays, SPSS 11.5 software was used for statistical analysis. Analysis of variance (ANOVA) followed by Duncan test was used to see the different amongst various groups. The significance level was set at *P*< 0. 05.

## Results

The results of antibacterial assay indicated that both flowers and leaves of *M. sylvestris *methanol extracts exhibited high bactericidal activity*. *The extracts were found to have strong antibacterial effects against *Erwinia carotovora*, a common plant pathogen bacteria, with MIC value of 128 and 256 µg/ml for the flowers and leaves extracts, respectively. The methanol extract of the plant flowers showed high antibacterial effects against some human pathogen of bacteria strains such as *Staph. aureus*, *Strep. agalactiae*, *Entro. faecalis*, with MIC value of 192, 200, and 256 µg/ml, respectively. Both flowers and leaves methanol extracts of the plant possessed modest antibacterial activity against other tested microorganism with MIC value range of 320-800 µg/ml (Table 1).

On the other hand, antifungal assay showed that the methanol extract of the plant flowers indicated modest antifungal activity. The extract inhibited the growth of *Sclerotinia sclerotiorum*, a plant pathogen fungus, and some human pathogen fungus like *C. kefyr *and *C. albicans* with MIC range of 640-800 µg/ml (Table 1). 

No considerable antibacterial and antifungal activity were found for the plant n-hexane and dichloromethane extracts.

**Figure 1.**
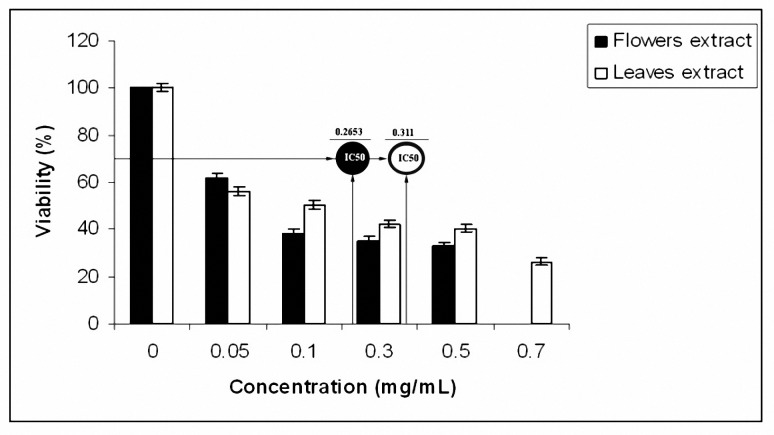
Cytotoxic effects of methanol extract of *M. sylvestris *on MacCoy cells. Each bar represents standard error of the mean

Our finding also showed that the plant methanol extracts exhibited rather high cytotoxic activity against McCoy line. The flower and leaves extracts reduce the viability of McCoy cells with IC_50_ value of 265.3 and 311.0 µg/ml, respectively (Figure 1). 

On the other hand, we did not find considerable phytotoxic activity for *M. sylvestris *extracts. This finding revealed that there is no significant differences in seed geremination between seed treated with the different concentration of the plant extracts and control test in seed germination (Table 2).

**Table 1.** Antimicrobial effects of methanolic extracts of *Malva sylvestris*

Treatment							
	Leaf extract (1500 µg)		Flower extract (1500 µg)		Erythromycin (30 µg)	Gentamicin (3 µg)	Amphotericin (10µg)
Microorganism							
	IZ (mm)	MIC (µg/ml)	IZ (mm)	MIC (µg/m)	IZ (mm)	IZ (mm)	IZ (mm)
*Escherichia coli*	640	32.0	512	31.0	16.0	20.0	-
*Staphylococcus aureus*	320	36.0	192	39.0	19.0	20.0	-
*Entrococcus faecalis*	340	35.0	256	38.0	21.0	16.0	-
*Streptococcus agalactiae*	320	35.6	200	38.0	21.0	16.0	-
*Erwinia carotovora*	256	37.0	192	39.0	21.0	19.0	-
*Staphylococcus aureus *)E_38_(	320	36.0	655	39.0	-		34.0
*Candida kefyr*	800	28.0	640	30.0	-	-	29.0
*Candida albicans*	800	26.5	640	28.0	-	-	24.0
*Aspergillus niger*	950	19.0	800	20.0	-	-	23.0
*Penicillum sp*	950	12.0	800	20.0	-	-	28.0
*Sclerotinia sclerotiorum*	950	19.0	800	21.0	16.0	20.0	-

IZ= Inhibition zone

MIC= Minimum inhibitory concentration

**Table 2.** Phytotoxic effects of *Malva* *sylvestris* leaves extracts

Concentration )mg/ml)	Germination(%)				
	Hex		DCM		Met
0	98 ± 2.3 a	92 ± 5.3 a		90± 3.8 a	
0.001	96 ± 3.1 a	98 ± 3.2 a		96 ± 2.2 a	
0.01	96 ± 2.2 a	96 ± 1.4 a		92 ± 3.2 a	
0.1	98± 3.3 a	92 ± 1.5 a		94 ± 2.5a	
1	92 ± 2.4 a	94 ± 1.8 a		90 ± 23.6a	

Mean values in the same column followed by the same letter are not significantly different at the 0.05 level according to the Duncan test. Hex: n-hexane extract, DCM: dichloromethane extract, Met: methanol extract

## Discussion

A literature study reported various pharmacological properties for *M. sylvestris* and other species of *Malva.* It is well-known that *M. sylvestris* can be utilized as an anti-inflammatory substance for the respiratory tract, GI tract, and the skin (11). The plant can be used topically or in a bath to treat abscesses, bruises, burns, dermatitis, swellings, and various ulcers (12,13). 

It is assumed that this pharmacological and biological activity of the plant should be attributed to presence of anthocyanidines, naphthaquinones, flavonoides or mucilaginous polysaccharides that are in high amounts in the plant fruits, flowers, leaves, and roots. 

A naphtaqiunone, namely, malvone A has been reported from stems of *M. sylvestris*. It was also shown that malvone A was regarded as a phytoalexin and was induced by some plant pathogen microorganisms (4). Therefore, this compound could be responsible for high antimicrobial activity of the *M. sylvestris* against different plant and human pathogen microorganisms. Therefore, the* M. sylvestris* extracts could be a good candidate for making a biopesticide. Nowadays, in spite of the successful pest control achieved with synthetic pesticides, the use of these chemicals has negative effects on environments and human being. Therefore, the use of the natural compounds for pest control might be one of the alternatives for environmentally friendly agriculture.

Our results revealed that *M. sylvestris *possesses antibacterial effects on methicillin resistant strain of *staph. aureus* (E38). At the recent years, the development of bacterial resistance to presently available antibiotics has caused a serious problem for global hygienic and health programs. This problem has necessitated the search for new antimicrobial agent with natural navigate.

The results herein reported, showed that *M. sylvestris* extracts exhibits high cytotoxic activity. Therefore, this plant could be considered as an antiproliferative agent. The plant has a rich ethnomedicinal history and has been used since ancient Greece and Roman times. The above ground portions of the plant have been used in pancakes and salads, cooked as greens and used as stuffing in some countries. The immature fruits may be consumed raw as a snack, as well (14). Therefore, high consumption of the plant leaves, flowers or fruits could be associated with a reduced risk of cancer.

## Conclusion

It was concluded that *M. sylvestris* indicated considerable bioactivities. This Iranian native plant can be used as an antiseptic agent to eliminate antibiotic resistance microorganisms. 
